# A Korean Family Presenting with Renal Cysts and Maturity-Onset Diabetes of the Young Caused by a Novel In-Frame Deletion of *HNF1B*

**DOI:** 10.3390/ijms25189823

**Published:** 2024-09-11

**Authors:** Ji Yoon Han, Jin Gwack, Tae Yun Kim, Joonhong Park

**Affiliations:** 1 Department of Pediatrics, College of Medicine, The Catholic University of Korea, Seoul 06591, Republic of Korea; han024@catholic.ac.kr; 2 Department of Preventive Medicine, Jeonbuk National University Medical School, Jeonju 54907, Republic of Korea; gwackjin@jbnu.ac.kr; 3 Research Institute of Clinical Medicine of Jeonbuk National University-Biomedical Research Institute of Jeonbuk National University Hospital, Jeonju 54907, Republic of Korea; 4 Department of Thoracic and Cardiovascular Surgery, Jeonbuk National University Medical School and Hospital, Jeonju 54907, Republic of Korea; 5 Department of Laboratory Medicine, Jeonbuk National University Medical School and Hospital, Jeonju 54907, Republic of Korea

**Keywords:** hepatocyte nuclear factor-1-beta, *HNF1B*, maturity-onset diabetes of the young, renal cyst, hepatic cyst, clinical exome sequencing

## Abstract

Maturity-onset diabetes of the young (MODY; OMIM # 606391) comprises a cluster of inherited disorders within non-autoimmune diabetes mellitus (DM), typically emerging during adolescence or young adulthood. We report a novel in-frame deletion of *HNF1B* in a family with renal cysts and MODY, furthering our understanding of *HNF1B*-related phenotypes. We conducted sequential genetic testing to investigate the glucose intolerance, renal cysts, hepatic cysts, and agenesis of the dorsal pancreas observed in the proband. A comprehensive clinical exome sequencing approach using a Celemics G-Mendeliome Clinical Exome Sequencing Panel was employed. Considering the clinical manifestations observed in the proband, gene panel sequencing identified a heterozygous *HNF1B* variant, c.36_38delCCT/p.(Leu13del) (reference transcript ID: NM_000458.4), as the most likely cause of MODY in the proband. The patient’s clinical presentation was consistent with MODY caused by the *HNF1B* variant, showing signs of glucose intolerance, renal cysts, hepatic cysts, and agenesis of the dorsal pancreas. Sanger sequencing confirmed the same *HNF1B* variant and established the paternally inherited autosomal dominant status of the heterozygous variant in the patient, as well as in his father and sister. The presence of early-onset diabetes, renal cysts, a family history of the condition, and nephropathy appearing before or after the diagnosis of diabetes mellitus (DM) suggests a diagnosis of *HNF1B*-MODY5. Early diagnosis is crucial for preventing complications of DM, enabling family screening, providing pre-conceptional genetic counseling, and monitoring kidney function decline.

## 1. Introduction

Maturity-onset diabetes of the young (MODY; OMIM # 606391) comprises a cluster of inherited disorders within non-autoimmune diabetes mellitus (DM), typically emerging during adolescence or young adulthood. Thus far, 14 causative genes linked to MODY have been identified, including *ABCC8*, *APPL1*, *BLK*, *CEL*, *GCK*, *HNF1A*, *HNF1B*, *HNF4A*, *INS*, *KCNJ11*, *KLF11*, *NEURO1*, *PAX4*, and *PDX1* [1]. *HNF1B*-associated MODY accounts for approximately 6% of all MODY cases, characterized by declining insulin secretion leading to progressive hyperglycemia [2]. Among the above genes, the hepatocyte nuclear factor 1β (*HNF1B*) gene, also known as transcription factor-2 (*TCF2*), is involved in the development of a specialized epithelium during both the early and late phases of embryogenesis, regulating the cell cycle. Mutations in the hepatocyte nuclear factor-1B (*HNF1B*) gene were first reported by Horikawa et al. in 1997 as a rare genetic cause of MODY associated with non-diabetic nephropathy [3]. Over 200 *HNF1B* mutations have since been identified, with the kidney and pancreas being the most commonly affected organs, leading to MODY and renal abnormalities. Renal cysts are the predominant manifestation among *HNF1B*-associated kidney diseases, though other renal anomalies, such as a single kidney, renal hypoplasia, and renal dysfunction, including hypomagnesemia or hyperuricemia, may also occur [4]. Extra-renal phenotypes include MODY, pancreatic hypoplasia or atrophy, genital tract anomalies, elevated liver enzymes, and early-onset gout. De novo *HNF1B* mutations account for approximately 50–60%, while the remainder are inherited in an autosomal dominant pattern [5,6]. Complete gene deletions on 17q12 are responsible for 50% of cases, while heterozygous mutations in the coding region or splice site of *HNF1B* constitute the remaining 50%, indicating haploinsufficiency as the disease mechanism. Here, we report a novel in-frame deletion of *HNF1B* in a family with renal cysts and MODY, furthering our understanding of *HNF1B*-related phenotypes.

## 2. Case Presentation

### 2.1. The Proband

**Figure 1:**
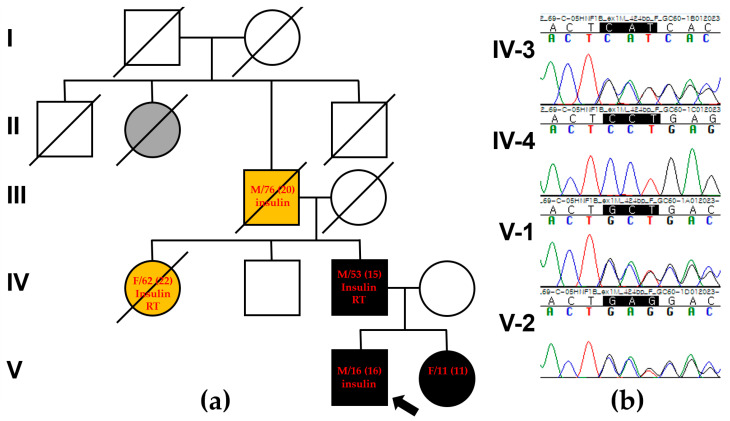
Pedigree analysis and segregation analysis. (**a**) The family pedigree shows autosomal dominant maturity-onset diabetes of the young (MODY). The proband is indicated by a black arrow. Numbers in parentheses indicate the age (year) at diagnosis. The gray symbol indicates that the family member was clinically suspected to have hereditary MODY, but this was not confirmed genetically. The yellow symbol indicates that the family member was clinically suspected to have diabetic triopathy, but this was not confirmed genetically. Black symbols indicate family members who were clinically suspected and confirmed by clinical exome sequencing. Treatments, including insulin and renal transplantation (RT), are described in each symbol. (**b**) Sanger sequencing confirmed a heterozygous *HNF1B* variant, c.36_38delCCT /p.(Leu13del), occurring as a consequence of autosomal dominant paternal origin in the proband (V-1) (Reference transcript ID: NM_000458.4).

**Figure 2:**
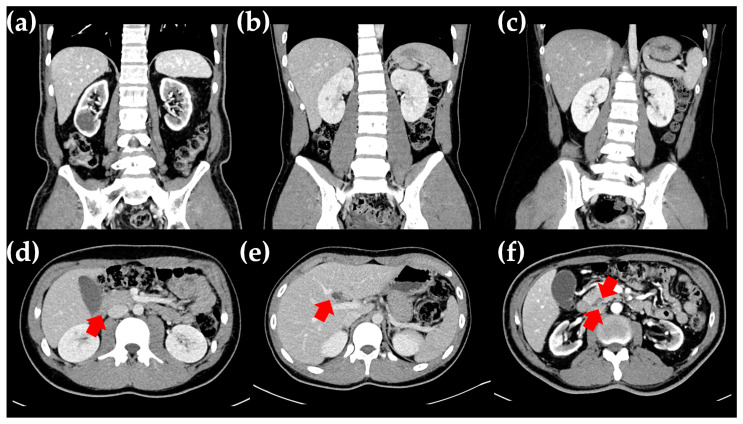
Radiologic findings in the proband and his family. (**a**–**c**) Abdominal CT findings show renal cysts in the proband’s father (**a**), the proband (**b**), and the proband’s sister (**c**). (**d**–**f**) The cortical thinning of both kidneys and atrophic changes in the pancreas (**d**) were found in the proband’s father, while a hepatic cyst (**e**) and agenesis of the dorsal pancreas (**f**) were found in the proband. The tail of the pancreas was not visible on consecutive axial slices, showing only the head and body of the pancreas (indicated by red arrow).

A 16-year-old Korean boy (V-1 in Figure 1a) presented at the Department of Pediatrics, Daejeon St. Mary’s Hospital (Daejeon, Republic of Korea), with a history of polyuria and polydipsia over the past month. He was the first child of non-consanguineous parents, and the pregnancy was uneventful. His growth percentiles were normal at birth. There was a family history of DM suggestive of an autosomal dominant pattern. Specifically, his grandfather (III-1 in Figure 1a) was diagnosed with DM in his late 20s, began dialysis in his 60s, and passed away at 70 due to a cerebrovascular event. His great-grandaunt (II-2 in Figure 1a) was diagnosed with DM in her 20s and passed away in her 30s due to a traffic accident. His aunt (VI-1 in Figure 1a) was diagnosed with DM at the age of 20, started dialysis at 50, and passed away at 55 due to sepsis as a complication of DM. The patient was 167 cm tall, weighed 63 kg, and had a BMI of 22.6 kg/m^2^ (60th percentile). Physical and neurological examinations revealed no abnormalities. His fasting glucose level was 301 mg/dL, and glycated hemoglobin (HbA1c) was 15.4%. Serum ketone bodies were elevated, and urine sugar was strongly positive. Autoimmune antibodies, including anti-GAD, anti-IAA, and anti-ICA, used for differentiating type I DM, were all negative. C-peptide and insulin levels were 2.13 ng/mL (normal range, 1.1–4.4) and 8.50 μU/mL (normal range, 1.90–23), respectively. Abdominal computed tomography (CT) revealed bilateral renal cysts, a hepatic cyst, and agenesis of the dorsal pancreas, with no abnormalities detected in other abdominal organs (Figure 2b,e,f). Based on clinical, imaging, and biological variables, MODY5 was suspected due to young-onset DM, normal BMI, renal cysts, insulin secretion dysfunction, negative autoantibodies to IA-2, and an HNF1B score of 14, which is a pivotal tool for rational genetic testing [7]. He was treated with intensified insulin therapy (total 0.2 units/kg), leading to improved glycemic control. He showed no signs of diabetic retinopathy, neuropathy, or nephropathy. 

### 2.2. The Proband’s Father

The proband’s father (IV-3 in Figure 1a), a 53-year-old man, was diagnosed with DM at the age of 15, requiring insulin injections thereafter. At the time of diagnosis, his fasting glucose level was 398 mg/dL, and his HbA1c was 17%. He was diagnosed with autoantibody-negative type I DM. Around the age of 35, he developed diabetic triopathy (retinopathy, nephropathy, and neuropathy), undergoing vitreoretinal surgery at 39. His renal function progressively declined, leading to end-stage renal disease in his late 40s, necessitating renal transplantation at 50. An abdominal CT scan revealed bilateral parenchymal thinning with cysts in both kidneys and pancreatic atrophy extending from the neck to the tail (Figure 2a,d). An ultrasound performed at age 39 showed a right renal cyst with a diameter of approximately 1 cm. However, a CT scan conducted at age 50 revealed that the cyst had enlarged to about 3 cm, accompanied by progressive renal atrophy.

## 3. Genetic Testing

Sequential genetic testing was conducted to explore the glucose intolerance, renal cysts, hepatic cysts, and agenesis of the dorsal pancreas observed in the proband. Initial evaluations, including conventional karyotyping and chromosomal microarray analysis, did not reveal any pathogenic structural or numerical chromosome changes or copy number variations. Next, we employed a comprehensive clinical exome sequencing (CES) approach using the Celemics G-Mendeliome Clinical Exome Sequencing Panel (Celemics, Inc., Seoul, Republic of Korea). This panel encompasses around 7000 genes associated with significant Mendelian genetic disorders, covering all pertinent regions. Massively parallel sequencing was performed with the DNBSEQ-G400RS High-throughput Sequencing Set and DNBSEQ-G400 sequencer (MGI Tech Co. Ltd., Shenzhen, China). Pathogenic variant interpretation adhered to the guidelines set by the American College of Medical Genetics and Genomics (ACMG) and the Association for Molecular Pathology (AMP). To identify potentially harmful variants, we used the following criteria: (1) variants located near or within exons of protein-coding genes linked to Mendelian diseases; (2) variants with allele frequencies less than 0.01; (3) variants causing nonsynonymous or nonsense changes in codons within exons, affecting highly conserved splice sites, or inducing frameshift mutations; (4) de novo or rare heterozygous, compound heterozygous, or homozygous variants in the same gene found in the proband; (5) genes included in the MODY Panel with clinical relevance, such as *APPL1*, *BLK*, *CEL*, *GATA6*, *GCK*, *HNF1A*, *HNF1B*, *HNF4A*, *INS*, *KCNJ11*, *KLF11*, *NEURO1*, *PAX4*, and *PDX1*; (6) the specific conditions of DM, glucose intolerance, renal cysts, hepatic cysts, and agenesis of the dorsal pancreas, which might be sporadic or inherited in an autosomal dominant manner, considering that the proband’s father and sister were affected. The allele frequencies of filtered variants were assessed using the Genome Aggregation Database (gnomAD, https://gnomad.broadinstitute.org/, accessed on 1 March 2024). In silico analyses were carried out to predict the pathogenicity of missense and insertion/deletion variants using MutationTaster (https://www.mutationtaster.org/, accessed on 1 March 2024) and VEST-4 (https://www.cravat.us/CRAVAT/, accessed on 1 March 2024).

**Figure 3:**
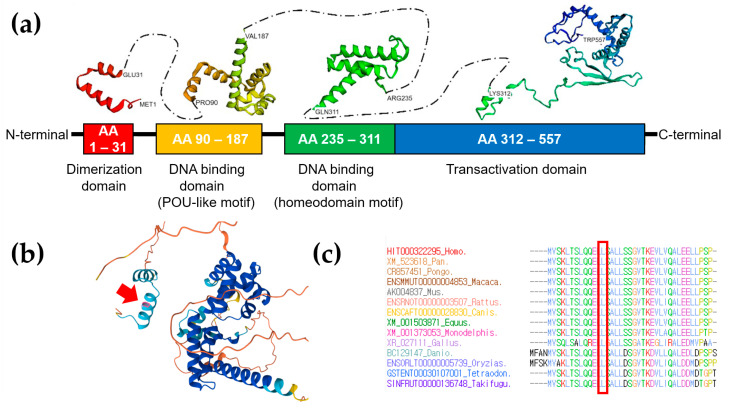
The 3D protein structure and conservation analysis of HNF1B. (**a**) The 3D protein modeling of the structure of human HNF1B using de novo protein modeling. The HNF1B protein structure was built through the DMPfold 1.0 Fast Mode algorithm on the PSIPRED server. RSCB PDB database; DNA-binding domain: 2DA6, 2H8R, and 5K9S. (**b**) Protein structure analysis using AlphaFold showed a very high per-residue confidence score (pLDDT) of 86.87 for the HNF1B p.Leu13 residue, highlighted in pink and indicated by a red arrow. (**c**) Sequence alignment of the conserved dimerization domain of the HNF1B protein in multiple vertebrate species. The protein sequence of the p.Leu13 residue is highly conserved between Homo sapiens and Takifugu. It is highlighted by the empty red box.

As a result, gene panel sequencing identified a heterozygous *HNF1B* variant, c.36_38delCCT/p.(Leu13del), as the most probable cause of MODY in the proband (Reference transcript ID: NM_000458.4). This in-frame variant was not present in the gnomAD database and was predicted to be “disease-causing” by MutationTaster and “deleterious” by VEST-4, with a VEST score of 0.75 (*p*-value of 0.03915). VEST assigns scores from 0 to 1, where a score of 1 indicates a high-confidence prediction of a functional mutation [8]. A potential splice effect was not detected by SpliceAI (https://spliceailookup.broadinstitute.org/, accessed on 1 March 2024). The clinical presentation of the patient was consistent with MODY caused by the *HNF1B* variant, including glucose intolerance, renal cysts, hepatic cysts, and agenesis of the dorsal pancreas, which is aligned with renal cysts and diabetes syndrome (OMIM #137920). Sanger sequencing confirmed that the c.36_38delCCT/p.(Leu13del) variant of *HNF1B* was linked to the phenotype and confirmed its autosomal dominant inheritance pattern. The variant was present in the patient, his father (IV-3), and his sister (V-2), but not in his mother (IV-4) (Figure 1b). This variant was classified as likely pathogenic based on ACMG guidelines, with criteria including PM2 (absent from Exome Sequencing Project, 1000 Genomes, or ExAC), PM4 (protein length changes due to in-frame deletions/insertions or stop-loss variants), PP1 (co-segregation with disease in multiple affected family members), and PP4 (the patient’s phenotype or family history specific to a single genetic cause). Protein structure analysis using AlphaFold (https://alphafold.ebi.ac.uk/, accessed on 1 March 2024) showed high per-residue confidence scores (90 > pLDDT > 70) of 86.87 for the HNF1B p.Leu13 residue in the dimerization domain (Figure 3a,b). Additionally, sequence alignment of the conserved dimerization domain of the HNF1B protein across various vertebrate species demonstrated that the p.Leu13 residue is highly conserved between humans and Takifugu (Figure 3c). Deletions and duplications were assessed using the multiplex ligation-dependent probe amplification assay with SALSA MLPA Probemix P241 MODY Mix 1 (MRC-Holland, Amsterdam, The Netherlands), and no abnormalities were detected except for c.36_38delCCT/p.(Leu13del).

## 4. Discussion

The *HNF1B* gene, located on chromosome 17q12, comprises an N-terminal dimerization domain, a homeobox and POU domain involved in DNA binding, and a transactivation domain at the C-terminus [9,10]. Belonging to the homeobox-containing family of transcription factors, *HNF1B* plays a crucial role in the development and function of epithelial tissues in the kidney, pancreas, liver, and genitourinary tracts [11]. Over 200 different variants have been reported within the *HNF1B* gene, with mutations primarily occurring in the first four exons encoding the dimerization and DNA-binding domains [12]. To date, the likely pathogenic or pathogenic variants registered in ClinVar comprise 80 frameshift, 78 missense, 38 nonsense, and 28 splice-site mutations (https://www.ncbi.nlm.nih.gov/clinvar/, accessed on 7 August 2024). The variable phenotypes observed among individuals or families, along with incomplete penetrance, pose challenges in understanding the condition, particularly due to the 50–60% rate of de novo mutations identified in index cases [13,14]. The majority of (likely) pathogenic variants in *HNF1B* are truncating (59.4%; 114/194), whereas missense variants, which cluster in important protein domains, constitute the second-largest group (39.1%; 75/194) [15]. In general, an out-of-frame mutation caused by deleting one or two nucleotides from a defined reading frame creates an entirely new open reading frame with completely different nucleotide triplets or codons. This usually results in a completely altered amino acid sequence, leading to a non-functional protein. In contrast to out-of-frame deletions (or insertions) of three nucleotides, in-frame deletions (or insertions) do not create a premature stop codon. This results in a protein with only a few amino acids added or missing, making it more likely that the protein remains functional. However, the prenatal and postnatal phenotypes of seven individuals with renal cysts caused by a novel in-frame deletion, p.(Gly239del), within the HNF1B DNA-binding domain were previously reported [15]. In our MODY5 family, an in-frame deletion of the *HNF1B* gene results in a protein lacking one amino acid, potentially affecting the tertiary structure of the protein. The deletion of the CCT removes the leucine amino acid but does not modify the reading frame, leaving the other amino acids unaltered. The in-frame deletion of leucine at position 13 identified in our case is highly conserved among different species. This deletion is located within the HNF1B dimerization domain, which mediates DNA dimerization. Even though in silico analysis predicted our in-frame deletion to have a harmful effect, additional functional studies are needed to demonstrate the functional impairment of the in-frame deletion. In particular, AlphaFold does not account for interactions with other molecules, such as nucleic acids, small-molecule co-factors, ions, and other non-protein components. Additionally, AlphaFold is not designed to model post-translational modifications or the structures of free nucleic acids. However, it may still predict a protein’s conformation as if it were bound to a ligand or ion, even if the actual ligand or ion is absent.

On the other hand, HNF1B is broadly expressed in various fetal tissues and is essential for visceral endoderm specification [16]. In animal models, including adult mice and rats, HNF1B is expressed in the liver, kidneys, pancreatic islets, stomach, and intestine [17]. Congenital anomalies of the female reproductive organs, such as bicornuate uterus, uterus didelphys, rudimentary uterus, and vaginal atresia, have also been reported [18]. Its expression during early embryonic development is critical for kidney and pancreas formation [19]. *HNF1B* plays a critical role in the growth of the collecting ducts, renal pelvis, and ureter, as well as in the differentiation of the metanephric mesenchyme, which are essential for nephron and collecting system development [20]. Regarding clinical findings, about 66% of patients with MODY5 have a family history of DM, and the median age at diabetes diagnosis is 16 years old. Renal cysts are present in about 72% of patients, hypomagnesemia in about 92%, and pancreatic hypoplasia in about 72% [21]. Various types of *HNF1B* variants have been reported, including 117 missense variants, 98 nonsense variants, 58 small deletions or insertions, and 24 splicing variants, according to the Human Gene Mutation Database. Most of these variants are located in the first four exons of the gene, with exons 2 and 4 and the intron 2 splicing site being hotspots. The highly conserved DNA-binding domain of *HNF1B* is crucial for transcription regulation [22]. Up to 50% of MODY5 cases are caused by a 17q12 deletion encompassing 15 genes, including *HNF1B*, and following deletions, missense or nonsense mutations are the most frequent [4,23]. Compared to deletions and mutations, *HNF1B* gene deletion is associated with significantly lower magnesium and serum creatinine levels and higher eGFR [24,25]. 

Pancreatic atrophy is reported in approximately 30% of patients, and DM in about 50% [26]. Imaging studies have shown dysgenesis of the pancreas, particularly in the body or tail, in approximately 70% of cases [27]. MODY typically develops before 25 years of age, and in this family, DM was diagnosed from adolescence to early adulthood. HbA1c levels ranged from 6% to 10%, and fasting blood glucose levels were around 500 mg/dL at diagnosis. Autoimmune antibodies were negative in all patients, and BMI was within the normal range, without obesity, in a previous study [28]. Renal dysfunction predominantly manifests as a chronic tubulointerstitial pattern and is often found in advanced chronic kidney disease (CKD, stages III–IV) [29]. Hypomagnesemia and hypokalemia are commonly observed, occurring in 10–62% and 46% of patients, respectively [30]. Liver dysfunction, characterized by an asymptomatic elevation in liver enzyme levels, is common in patients with *HNF1B* mutations [31]. However, structural liver abnormalities have been observed in around 30% of patients with *HNF1B* variants [24]. In this family, renal cysts were present, and renal dysfunction progressed slowly. Hypomagnesemia, hyperuricemia, liver dysfunction, and genital abnormalities were not observed. Dorsal pancreatic agenesis is extremely rare but may be present in a significant portion of DM cases, potentially up to 50%. The proband was diagnosed with DM and revealed pancreatic dysgenesis at 16 years old. Other acquired causes are not suspected, and *HNF1B* is known to be involved in pancreatic organogenesis. However, in his 53-year-old father, diffuse atrophic changes in the pancreas may be a secondary finding of DM or a manifestation of an *HNF1B* mutation. Since the proband and his sister differ in sex and age, variations in symptoms, such as BMI and the presence or absence of pancreatic abnormalities, are expected. Long-term follow-up is essential to evaluate whether their clinical symptoms diverge over time.

Among patients with congenital abnormalities of the kidney and urinary tract, approximately 10% had *HNF1B* mutations. Predictive factors for detecting *HNF1B* mutations included bilateral renal anomalies, renal cysts of unknown origin, a combination of two major renal anomalies, and hypomagnesemia. In this family, clinical manifestations suggested a moderate predictive value for *HNF1B* mutations prior to other genetic tests. However, the patients’ main concern was early-onset diabetes mellitus with an autosomal inherited pattern, which led us to conduct broader genetic testing, including genes associated with MODY. Most patients with *HNF1B*-related nephropathy typically present with simple renal cysts without a significant decrease in kidney function. Renal dysfunction usually manifests in the 40s, and approximately 3-50% of patients progress to chronic kidney disease (CKD), ultimately requiring renal transplantation, particularly in cases without diabetic nephropathy [32,33,34,35]. Generally, *HNF1B*-related nephropathy exhibits a slow progressive course in childhood, except for very early onset cases. Patients with *HNF1B* haploinsufficiency may present with renal hypoplasia and dysgenesis, familial juvenile hyperuricemic nephropathy, glomerulocystic kidney disease, and renal interstitial fibrosis, ultimately leading to CKD [26]. Less than 1% of affected patients with *HNF1B* mutations exhibit DM alone [36]. Studies involving reverse-transcription PCR and in situ hybridization have shown that HNF-1beta mRNA is observed in normal human metanephrons, with the highest transcript levels observed in fetal medullary and cortical collecting ducts and lower levels in nephrogenic cortex mesenchyme, primitive nephron tubules, and immature glomeruli [37]. In animal models, the inactivation of *HNF1B* from postnatal day 10 onward does not result in cystic dilations in tubules after their proliferative morphogenetic extension is completed [38]. *HNF1B* directly regulates the transcription of Pkhd1, and inhibition of *PKHD1* gene expression may affect the development of renal cysts in humans with *HNF1B* mutations [39].

DM, the second most common feature of *HNF1B* defects, has been observed in 5–50% of *HNF1B* mutation carriers in previous studies, accounting for approximately 5% of cases of MODY [40,41,42,43]. *HNF1B* is a tissue-specific transcription factor that forms homodimers or heterodimers with HNF1A and transactivates various genes, including albumin, alpha-fetoprotein, and glucose transporter 2 [44,45]. Defects in *HNF1B* lead to alterations in pancreatic morphology, ranging from pancreatic dysgenesis to diffuse pancreatic atrophy [46]. Pancreatic exocrine deficit has been reported in 20–75% of patients, and MODY is caused by beta-cell dysfunction and insulin resistance [47]. Evidence of pancreatic exocrine dysfunction has been observed in patients, as evidenced by fecal elastase deficiency [48]. Patients with *HNF1B*-MODY often respond poorly to oral hypoglycemic agents such as sulfonylureas, with early insulin therapy required in up to 80% of cases [46,49,50]. In patients with *HNF1B*-MODY, renal involvement is not associated with diabetic nephropathy but rather with aberrant embryonic development [51]. Long-term DM complications, including retinopathy and nephropathy, occur in patients with the longest duration of DM (median 21 years) [52]. In animal models, a precursor cellular stage of the embryonic pancreas and *HNF1B* in a genetic hierarchy control the formation of pancreatic endocrine cells [53]. *HNF1B* is targeted by miR-802-dependent silencing, and it has been revealed that a short hairpin RNA (shRNA)-mediated reduction in *HNF1B* in the liver leads to glucose intolerance, damage to insulin signaling, and increased hepatic gluconeogenesis. Conversely, hepatic overexpression of *HNF1B* enhances insulin sensitivity in Lepr(db/db) mice [54]. Our familial cases were diagnosed between ages 15 and the 20s, presenting with blood glucose levels around 300 mg/dL at diagnosis, accompanied by markedly reduced insulin secretion. Uric acid and magnesium levels were initially within normal ranges but increased around the time of dialysis initiation. Other studies similarly reported comparable blood glucose and insulin secretion patterns but varied in their findings for uric acid and magnesium levels [55]. Pancreatic developmental abnormalities were observed only in the proband, consistent with the known influence of the *HNF1B* gene on pancreatic development.

On the other hand, patients with *HNF1B* mutations often exhibit prevalent liver enzyme dysfunction, particularly in association with MODY5, affecting around 40% of adults [13]. Histopathological findings typically reveal bile ductopenia, steatosis, and periportal fibrosis, which may result in neonatal or adult cholestatic hepatopathy [56]. While other liver abnormalities have not been commonly reported, the proband in this family presented with a single hepatic cyst but normal liver function. Liver involvement in *HNF1B*-related conditions has not been extensively studied, with reported cases mainly presenting as an asymptomatic elevation in transaminase levels or, less commonly, as cholestatic liver diseases. Unusually, the proband in our family had a simple hepatic cyst without abnormal liver enzymes or related symptoms. These findings contribute to expanding our understanding of *HNF1B*-MODY5. Further studies are necessary to establish the underlying mechanisms and genotype–phenotype correlations. Clinical manifestations in our family presenting with renal cysts and MODY caused by the *HNF1B* c.36_38delCCT /p.(Leu13del) variant are summarized in Table 1.

## 5. Conclusions

In conclusion, we have described a family with MODY5 characterized by a novel in-frame deletion mutation affecting the dimerization domain of the *HNF1B* gene. Notably, contrary to previous reports, the proband exhibited a single hepatic cyst, with liver enzyme levels within normal ranges. The presence of early-onset diabetes, renal cysts, a family history, and nephropathy appearing before or after the diagnosis of DM suggests a diagnosis of *HNF1B*-MODY5. Early diagnosis is crucial for preventing complications of DM and allows for family screening and pre-conceptional genetic counseling. Patients with *HNF1B*-MODY should receive comprehensive medical care from endocrinology, nephrology, urology, and gynecology departments. To date, there is no well-established genotype–phenotype correlation indicating which types of variants are associated with particular clinical manifestations. The factors contributing to phenotypic variation remain inadequately understood and could potentially include the functional consequences of distinct gene variants, stochastic fluctuations in *HNF1B* gene expression during the early developmental period, or additional genetic and/or environmental influences. The current findings highlight the need for further investigation through prospective studies.

## Figures and Tables

**Table 1:** Clinical manifestations in a family presenting with renal cysts and maturity-onset diabetes of the young caused by the *HNF1B* p.Leu13del variant.

Patients	Proband’s Father (VI-3)		Proband (V-1)	Proband’s Sister (V-2)	Reference Ranges
**Sex/age (year)**	M/53		M/16	F/11	
**BMI (** **kg/m^2^)**	22.5		22.6	28.4	18.5–22.9
**HNF1B score**	14		14	10	<8
**Renal cyst**	bilateral		bilateral	bilateral	
**Pancreatic abnormalities**	Diffuse atrophic changes		Agenesis of dorsal pancreas	none	
**Genitourinary tract defect**	none		none	none	
**Laboratory findings at age**	at 15 y old	at 50 y old	at 16 y old	at 11 y old	
**FBS (mg/dL)**	398	228	301	115	1–120
**HbA1c (%)**	18	7.5	15.4	6.1	4.5–5.6
**BUN (mg/dL)**	15	28.9	13.9	13.9	6–20
**Creatinine (mg/dL)**	1	5.52	1.03	0.58	0.5–1.2
**eGFR (mL/min per 1.73 m^2^)**	115	10.8	107	141	90–120
**AST/ALT (IU/L)**	15/21	16/14	25/12	17/15	8–40/5–41
**Uric acid (mg/dL)**	2.4	3.9	2	6	2.4–7
**Mg (mg/dL)**	1.5	2.3	1.6	1.8	1.6–2.6
**Ketone body (mg/L)**	380.10	27.1	371.30	120	0–120
**Ketone**	negative	negative	negative	negative	negative
**Autoantibodies**	negative	n/a	negative	negative	negative
**Insulin (AC) (uU/mL)**	0.90	n/a	0.80	1.5	1.9–23
**Insulin (PC) (uU/mL)**	8	n/a	8.5	10	1.9–23
**C-peptide (AC) (ng/mL)**	0.8	1.65	0.75	1.4	1.1–4.4
**C-peptide (PC) (ng/mL)**	2	1.93	2.13	3.2	1.1–4.4
**Urine sugar (mg/100mL)**	3+	2+	3+	negative	negative
**Urine protein (mg/100mL)**	negative	2+	negative	negative	negative
**Urine albumin/Cr ratio (ug/mg)**	10	200	6	2	<30

BMI, body mass index; y, year; FBS, fasting blood sugar; eGFR, estimated glomerular filtration rate; +, positive urine dipstick testing; Cr, creatinine.

## Data Availability

Data are contained within the article.
